# Measuring objective fatigability and autonomic dysfunction in clinical populations: How and why?

**DOI:** 10.3389/fspor.2023.1140833

**Published:** 2023-03-30

**Authors:** Guillaume Y. Millet, Mathilde F. Bertrand, Thomas Lapole, Léonard Féasson, Vianney Rozand, David Hupin

**Affiliations:** ^1^ Université Jean Monnet Saint-Etienne, Université Savoie Mont-Blanc, Inter-university Laboratory of Human Movement Biology, F-42023, Saint-Etienne, Lyon, France; ^2^ Institut Universitaire de France (IUF), Paris, France; ^3^ Service de physiologie clinique et de l'exercice, CHU de Saint-Étienne, Saint-Étienne, France; ^4^ Centre Référent Maladies Neuromusculaires rares - Euro-NmD, CHU de Saint-Étienne, Saint-Étienne, France; ^5^ Jean Monnet University Saint-Etienne, Mines Saint-Etienne, University hospital of Saint-Etienne, INSERM, SAINBIOSE, U1059, DVH team, Saint-Etienne, France

**Keywords:** fatigue, neuromuscular function monitoring, deconditioning, autonomic nervous system, heart rate variability, baroreflex, cardiopulmonary exercise testing

## Abstract

Fatigue is a major symptom in many diseases, often among the most common and severe ones and may last for an extremely long period. Chronic fatigue impacts quality of life, reduces the capacity to perform activities of daily living, and has socioeconomical consequences such as impairing return to work. Despite the high prevalence and deleterious consequences of fatigue, little is known about its etiology. Numerous causes have been proposed to explain chronic fatigue. They encompass psychosocial and behavioral aspects (e.g., sleep disorders) and biological (e.g., inflammation), hematological (e.g., anemia) as well as physiological origins. Among the potential causes of chronic fatigue is the role of altered acute fatigue resistance, i.e. an increased fatigability for a given exercise, that is related to physical deconditioning. For instance, we and others have recently evidenced that relationships between chronic fatigue and increased objective fatigability, defined as an abnormal deterioration of functional capacity (maximal force or power), provided objective fatigability is appropriately measured. Indeed, in most studies in the field of chronic diseases, objective fatigability is measured during single-joint, isometric exercises. While those studies are valuable from a fundamental science point of view, they do not allow to test the patients in ecological situations when the purpose is to search for a link with chronic fatigue. As a complementary measure to the evaluation of neuromuscular function (i.e., fatigability), studying the dysfunction of the autonomic nervous system (ANS) is also of great interest in the context of fatigue. The challenge of evaluating objective fatigability and ANS dysfunction appropriately (i.e.,. how?) will be discussed in the first part of the present article. New tools recently developed to measure objective fatigability and muscle function will be presented. In the second part of the paper, we will discuss the interest of measuring objective fatigability and ANS (i.e. why?). Despite the beneficial effects of physical activity in attenuating chronic fatigue have been demonstrated, a better evaluation of fatigue etiology will allow to personalize the training intervention. We believe this is key in order to account for the complex, multifactorial nature of chronic fatigue.

## Introduction

Chronic fatigue, defined as “*an unusual and persistent feeling of fatigue related to the disease or treatments that interfere with the person's usual functioning*” (1), is a major symptom in many diseases, and often the most common and debilitating one. Chronic fatigue is typically measured using subjective self-report tools such as the Functional Assessment of Chronic Illness Therapy-Fatigue (FACIT-F), the Modified Fatigue Impact Scale or the Checklist Individual Strength questionnaire. As many as 40 questionnaires have been validated to assess cancer-related fatigue alone (2). Persistent fatigue is an issue in most chronic diseases such as cancer, multiple sclerosis (3, 4), inflammatory bowel diseases (5), rheumatic diseases including fibromyalgia (6), chronic kidney disease (7) or chronic venous disease (8). Cancer patients reported that fatigue adversely affected their daily lives more than pain (9). Conversely, and quite surprisingly, oncologists believed that pain adversely affected their patients more than fatigue (9), highlighting the critical need to better consider fatigue as a major symptom in order to treat it. Depending on the disease and, within a disease, depending on the patient, its trajectory may vary, yet fatigue may last for an extremely long period. For instance, it has been shown that fatigue can persist for months or years following treatment in around one-third of patients diagnosed with cancer (10). Even more notable, our group observed that 57% of ICU survivors will suffer from fatigue from six months to five years after ICU discharge (11).

Chronic fatigue impacts quality of life, reduces the capacity to perform activities of daily living, and has socioeconomical consequences such as impairing return to work. For example, 5 years after therapy, only 51% (for females) and 63% (for males) of patients with Hodgkin lymphoma were working or were in professional education if they suffered from severe fatigue, compared with 78% and 90% if they did not (12). A survey conducted in the US in 2007 has reported that workers with fatigue cost employers 136 billion dollars annually in health-related lost productive time, an excess of 101 billion dollars compared with workers without fatigue (13). High levels of fatigue have even been associated with excess mortality in the general population (14). Despite the high prevalence and deleterious consequences of fatigue, little is known about its origin and contributing mechanisms. This is problematic since this lack of knowledge prevents clinicians to efficiently consider and treat this symptom (15). Numerous causes have been proposed to explain chronic fatigue. They encompass psychosocial and behavioral aspects (e.g., sleep disorders) and biological (e.g., inflammation), hematological (e.g., anemia) as well as physiological origins, the later referring to physical deconditioning. This prolonged or chronic fatigue is often pathological but can also affect healthy populations. Importantly, it must not be confounded with transient fatigue (16, 17), also known as neuromuscular fatigue or performance fatigability. It is defined in the present paper as a reduction of functional capacity and will be discussed in detail below. Discussing the factors potentially explaining chronic fatigue is beyond the purpose of the present paper. In this manuscript, we will focus on two particular factors: (i) a deteriorated resistance to objective fatigability (17) and (ii) the dysfunction of the autonomic nervous system (ANS) that plays a major role in cardiac and vascular adaptations to environmental stress (18). Autonomic functions may be useful as objective physiological markers for chronic fatigue because autonomic dysfunction can accentuate the pejorative prognostic of the disease (19). Assessing autonomic dysfunction in patients with fatigue aids targeting therapeutic interventions, in particular the development of management strategies by helping to tailor the amount of physical activity, as performed in athletes (20). Although objective fatigability and ANS may not be connected, they are both objective measures that can be associated with fatigue, explaining why they are associated in the present manuscript.

## Measuring objective fatigability resistance: How?

Even if the most used definition of fatigability is an exercise-induced reduction in maximal voluntary contraction (MVC), there is no clear consensus on the definition of objective fatigability, and this may ultimately affect its quantification and determinants (21). For instance, some have suggested that a reduction of accuracy, precision, or endurance, among other factors, may be considered as indices of objective fatigability. This section will describe the parameters that affect the evaluation of objective fatigability when defined as reduction of functional capacity, both the fatiguing task and the way fatigability (i.e. the final outcomes) is measured.

### Parameters of the fatiguing test

#### Workload

Although many exercise protocols have been used in the literature, for instance using maximal incremental test [e.g., (22)], the “classic” fatiguing protocols involve repetitions of sustained MVC [e.g., (23)] or repetitions of MVCs either in isometric [e.g., (24)] or isokinetic mode [e.g., (25)]. Then a fatigue index that consists in normalizing the last contractions to the initial ones is calculated. Alternatively, the fatiguing task involves sustained contractions at a given percentage of MVC, usually in isometric mode. The issue with these protocols is that fatigability is inversely correlated to maximal strength (26). Larger muscle mass and strength can play a role in limiting blood flow more rapidly in stronger subjects during low-to-moderate force sustained isometric contractions performed at the same relative intensity, as reported when comparing men and women (27). More problematic is the fact that normalization to a given percentage of MVC may give a false representation of the reality. For instance, Beretta-Piccoli et al. (28) recently compared objective fatigability of facioscapulohumeral muscular dystrophy (FSHD) patients to healthy controls during isometric sustained elbow flexions at 20% of their MVC for 2 min then at 60% MVC until exhaustion. They found that endurance time was greater and EMG indices of fatigability were less affected in FSHD patients than in controls. The explanation was related to the fact that MVC was 19.8 kg in FSHD patients vs. 28.8 kg in heathy subjects. In other words, if the fatiguing task was requesting an absolute level of force or a resistance scaled to body weight, the conclusion about endurance time and fatigability would have been completely opposite. This example shows that the type of workload used in the fatiguing tasks (percentage MVC, absolute value, percentage body weight, etc.) can dramatically change the conclusion about fatigability. Our group has recently used lower limbs fatiguing exercises scaled on body weight (29–32) since we believe that this is more representative of activities of daily living (walking, climbing stairs, etc.). By doing so, we were able to show that cancer patients (29, 30) and people with multiple sclerosis (31) who suffered from chronic fatigue had greater levels of objective fatigability than their non-fatigued counterparts.

#### Incremental vs. constant load

**Figure 1:**
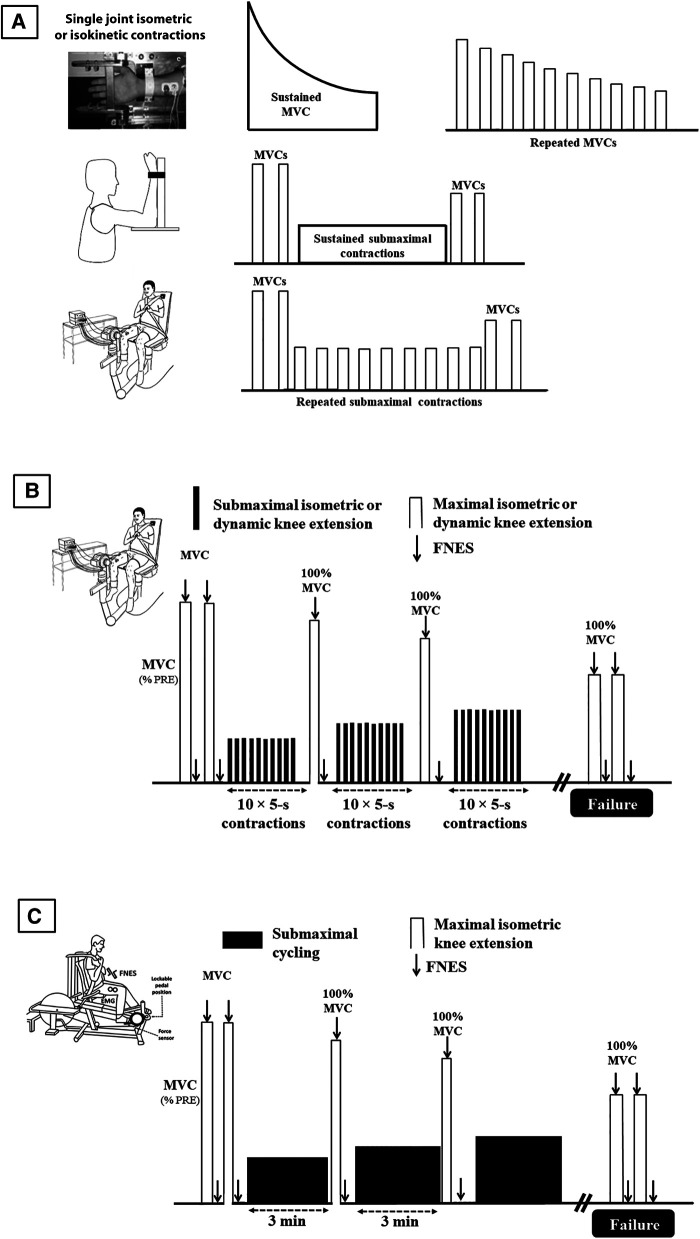
Main protocols used to assess objective fatigability, from non-specific evaluations (**A**), to more ecological ones (**B**), and to highest level of ecological validity (**C**). Lower limbs protocols also involve plantar flexor muscles, usually in isometric mode. MVC: maximal voluntary contraction; FNS: femoral nerve electrical stimulation.

As explained above, constant load protocols are probably the most used fatiguing designs in the literature. They consist in sustained contractions, either submaximal [e.g., (28) or maximal [typically 2-min MVC, e.g., (33)], or intermittent contractions that can again be maximal or submaximal (Figure 1A). When fatigability is only measured at task failure, it must be assumed that the motivation of the participant is maximal. Data showing that the variability in times to task failure is greater in clinical populations (34) suggest that it may less be the case than in healthy subjects. Furthermore, both clinical and healthy populations rarely reach exhaustion in activities of daily living, limiting the transfer of the results to ecological situations. To address these issues, we proposed a test based on incremental loading and regular assessments (from submaximal to exhaustion), i.e., a test that limits the influence of participant's cooperation and motivation: the quadriceps intermittent fatigue (QIF) test (35). The original QIF test involves measurements of isometric MVC and evoked knee extensors forces *via* femoral nerve stimulation before the fatiguing exercise, after each set of 10 submaximal isometric contractions (5-s on/5-s off, starting at 10% MVC with 10% increments) and at task failure (Figure 1B). By measuring voluntary and evoked forces at rest, at iso-workload and at task failure, this test assesses quadriceps strength (i.e., MVC), endurance (i.e., the total number of contractions) and fatigability (i.e., the progressive decline in MVC) in one session. The QIF test has already been used to assess various clinical populations such as patients with fibromyalgia syndrome (36), FSHD (37, 38) or Covid-19 ICU survivors (32).

#### Muscle mass and contraction mode

While in some rare protocols, fatigability was induced by electrostimulation [e.g., (39)], single-joint (mostly upper body) isometric voluntary contractions exercise is the standard task when designing a fatiguing exercise in clinical populations. For instance, Taul-Madsen et al. (40) reported that most studies (74%) investigating the underlying physiological mechanisms of fatigability in people with multiple sclerosis are performed in isolated hand models (41). The same probably applies to most clinical conditions. However, models involving the lower extremities might be more relevant for physical function and mobility, which is ranked as one of the most important bodily functions by patients together with pain and fatigue (42). Upper limbs are also mostly assessed in cancer (24, 43–47) despite some studies have investigated the ankle dorsiflexor (48) or the quadriceps (22, 29, 49, 50) muscles. The original QIF test has been designed using isometric task. Since then, it has been validated in concentric mode (51). It is important since the contraction mode plays a role in the conclusion made about differences in fatigability between populations. Our group published a meta-analysis showing that there was more objective fatigability (i.e., greater force decrease) in young subjects than in elderly when fatigability was induced by isometric tasks but not when the fatiguing exercise was performed in dynamic mode (52). Gaemelke et al. (53) compared isometric and concentric fatigability in people with multiple sclerosis and healthy subjects and found that objective fatigability did not differ between the two protocols. However, they reported that fatigability variance was mainly attributed to central deficit in the sustained isometric protocol and to both central and peripheral alterations in the concentric protocol. Yet in Varesco et al. (51) and Gaemelke et al. (53), the tests were in dynamic mode but still single-joint exercises, i.e., it does not involve large muscle mass recruited during most activities of daily living.

A dynamic fatiguing exercise where power output can be easily measured and that involves large muscle masses compared to single-joint isokinetic tasks is cycling. Compared to isolated muscle tasks, cycling increases the need to increase blood delivery to multiple, large muscles and stimulates the ventilatory response. Greater maximal cardiac output or greater stress on the respiratory function may become problematic for elderly population or patients with chronic obstructive pulmonary disease so that cardiac or lung functions may be the limiting factors in cycling but not during single-joint isometric exercise. This may partly explain why young subjects (25 years) performed 77% more work than their older (72 years) counterparts during cycling to exhaustion at 80% of peak power output whereas they performed “only” 33% more work during single-leg knee-extension (54). Also, in both groups, cycling induced less peripheral alterations at exhaustion than single-leg knee-extension (54), a result already found when comparing one vs. two legs knee extensions (55). This latter result suggests that beyond cardiorespiratory limitation, another explanation to the lower peripheral alterations using larger muscle mass is that single leg exercise may confine group III and IV skeletal muscle afferent feedback to a small muscle mass, enabling the central nervous system to tolerate greater peripheral alterations.

A typical setup in fatigue studies is to use the same ergometer to both induce and measure fatigue, e.g., sustained isometric contraction for a fixed duration or until task failure. To assess fatigue due to whole-body exercises, a solution is to fatigue subjects and measure their fatigability on two different ergometers (typically treadmill or ergocycle to fatigue the subject and quadriceps ergometer to measure objective fatigability). In that case, the patient must be transferred to the ergometer where fatigability is assessed. The delay usually taken for this transfer, typically 1 to 4 min (56–61), is problematic because subjects recover quickly after exercise, particularly after short and intense exercises. The delay taken to quantify objective fatigability may affect both central (33, 62) and peripheral (63) function. Not only the amplitude of fatigability (i.e., force loss) may be underestimated but fatigability etiology may not be properly evaluated. The reason is that the central nervous system recovers faster than muscle function (64) so that the role of central component is likely underestimated/missed. To address this issue, we designed a recumbent cycling ergometer that permits fatigability measurement within 1 s after cycling. The ergometer has instrumented pedals that can be locked instantly in a fixed position similar to what can be performed on an isometric chair. It was found to be as reliable as traditional measurements performed with an isometric chair (65). It allows to assess objective fatigability without any recovery period after a dynamic exercise with a large muscle mass (Figure 1C). As such it represents a major methodological advance. Since then it has been used in several studies with clinical populations (see below).

#### Other protocols used as a surrogate of fatigability measurements

Testing fatigability with clinical populations must be short since time for diagnosis is limited. For that reason, the six-minute walk test is sometimes used to study objective fatigability (by comparing the first and last minute or changes in spatiotemporal/kinematic parameters) for instance in people with multiple sclerosis or in older adults (66). Similarly, in spinal muscular atrophy, performance has been used as a surrogate of fatigability, performance being assessed during endurance shuttle tests, i.e., Nine Hole Peg Test and Box and Block Test for upper limbs and the endurance shuttle walk test for lower limbs (67). Although those tests provide relevant information about motor performance, they do not allow to directly assess fatigability [although they induce a decrease in functional capacity (67),] *per se* and they do not help to better understand its etiology. For instance, the six-minute walk test is too dependent on the energy cost of walking and pacing strategies. Other attempts have been made to measure fatigability in a real-life setting using wearable magneto-inertial sensor or simple wrist-worn accelerometer, sometimes coupled with assessment of subjective fatigue (68).

### Parameters of the objective fatigability measurements

**Figure 2:**
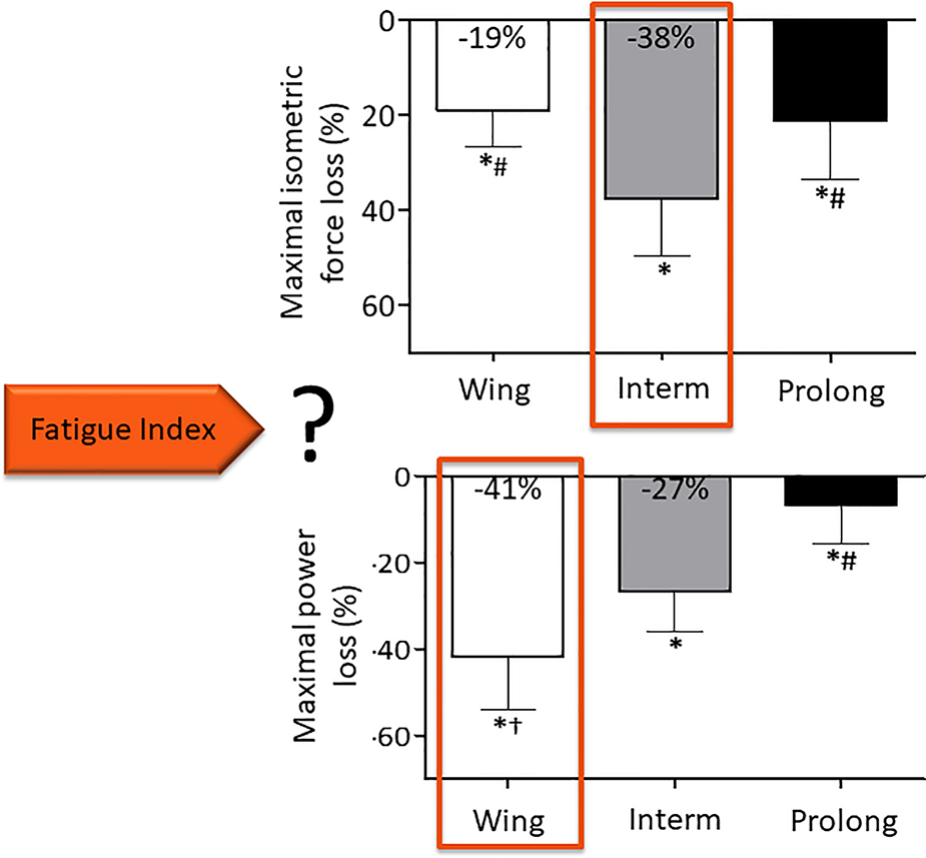
Neuromuscular fatigue depends on the index chosen. Adapted from (71). Wing: Wingate test, Interm: 10-min cycling exercise at a power output 5% above the respiratory compensation point, Prolong: 90-min cycling exercise at a power output 20% below the gas exchange threshold.

Although objective fatigability is classically measured as a reduction of maximal isometric force, the definition encompasses a decrease in maximal performance that could also be dynamic force or maximal power. It has been shown that isometric and dynamic measurements do not give the same results (69, 70). Our group recently assessed both MVC and maximal power (assessed during a 7-s sprint) after three different fatiguing cycling exercises: a Wingate test (30 s all out, maximal intensity), a 10-min cycling exercise at a power output 5% above the respiratory compensation point (intermediate duration and intensity) and a 90-min cycling exercise at a power output 20% below the gas exchange threshold (prolonged exercise) (71). Maximal power decreased relatively more than MVC force after the Wingate test whereas MVC force decreased more after the moderate intensity task. Thus, depending on the fatigue index considered, the response to the question about the most fatiguing exercise was totally different: it was the Wingate when maximal power was chosen but it was the intermediate duration and intensity task when MVC force was the selected index (Figure 2) (71). As highlighted in the meta-analysis described above (52), the discussion about which fatigue index, as well as the type of fatiguing exercise considered, is particularly relevant when exploring age-related changes in fatigability. Depending on the type of fatiguing tasks, there is no difference in the isometric force loss between young and old subjects or even less isometric MVC loss in elderly, yet maximal power decreases more in older adults (52). We concluded that the type of assessment (isometric force vs. power) must be considered in identifying age-related fatigability. The two approaches (i.e. measuring MVC vs. power reduction) are complementary and the researchers may want to use one or the other method, depending on the research question, or may sometimes want to assess both [e.g., (72)].

To investigate the causes of objective fatigability, evoked stimulations at cortical, cervicomedullary/thoracic or peripheral nerve levels are commonly used. These methods use artificial stimulations either superimposed to an MVC to assess maximal voluntary activation (any decrease in voluntary activation being an index of central alterations), or on relaxed muscles to assess peripheral changes due to exercise. Because those stimulations are uncomfortable, some adjustments of the protocols, for instance using magnetic instead of electrical stimulations, have been tried in patients but often without success because of the larger percentage body fat (73) or the reduced nerve excitability (37). The reader interested by this topic is invited to report to some of our previous review papers where the advantages and the limitations of these protocols are discussed (21, 74–76).

The classic evaluations of objective fatigability using MVC can hide some information about the actual state of the subjects. Indeed, it is possible to observe no decrease in MVC force despite there being some level of fatigue. This happens when low-frequency fatigue (77), defined as a preferential loss of force evoked by low-frequency stimulation as compared to loss of force evoked by high-frequency stimulation, occurs. Since motor units discharge at high rate during MVC, it may not decrease or have fully recovered despite the existence of this type of muscle fatigue. In order to encompass low-frequency fatigue, MacIntosh and Rassier (78) have proposed “a response that is less than the expected or anticipated contractile response, for a given stimulation” as the definition of objective fatigability. Recently, a new portable device allowing quadriceps low-frequency fatigue assessment in the field in only two minutes (Myocene®) has been commercialized and tested by our group against laboratory measurements after an eccentric exercise (79). Even though this device has mostly been used with athletes so far, it could potentially be used by clinicians in the future to assess persistent low-frequency fatigue in knee extensors.

The rate of force development (RFD), obtained from the ascending part of the force-time curve of an explosive contraction, has also been used to objectively assess fatigability. In their review, D'Emanuele et al. (80) reported that on average, peak RFD was more susceptible to exercise-induced fatigability than isometric MVC. These authors also suggested that a rationale for using RFD rather than MVC is often lacking in the studies that have used RFD and they recommended that RFD should be evaluated when measuring fatigability due to exercises based on relatively rapid contractions (80). The use of surface EMG to objectively measure fatigability has also been largely used [e.g., (45)], particularly through the analysis the frequency content, reporting the changes in mean or median frequency of the signal as fatigability outcomes. This will not be developed in the present paper as we believe it is of little clinical significance because of the time needed to install EMG electrodes, to analyze the data and to normalize the signal.

### Measuring autonomic dysfunction

ANS is a component of the peripheral nervous system that regulates the involuntary physiological process of our body, in particular involving blood pressure and heart rate. An ANS dysregulation i.e., increased activity of sympathetic tone and/or less parasympathetic tone at rest reveals a body homeostasis dysfunction and can predict cardiovascular and metabolic illnesses and is directly correlated with mortality in several diseases (81).

**Figure 3:**
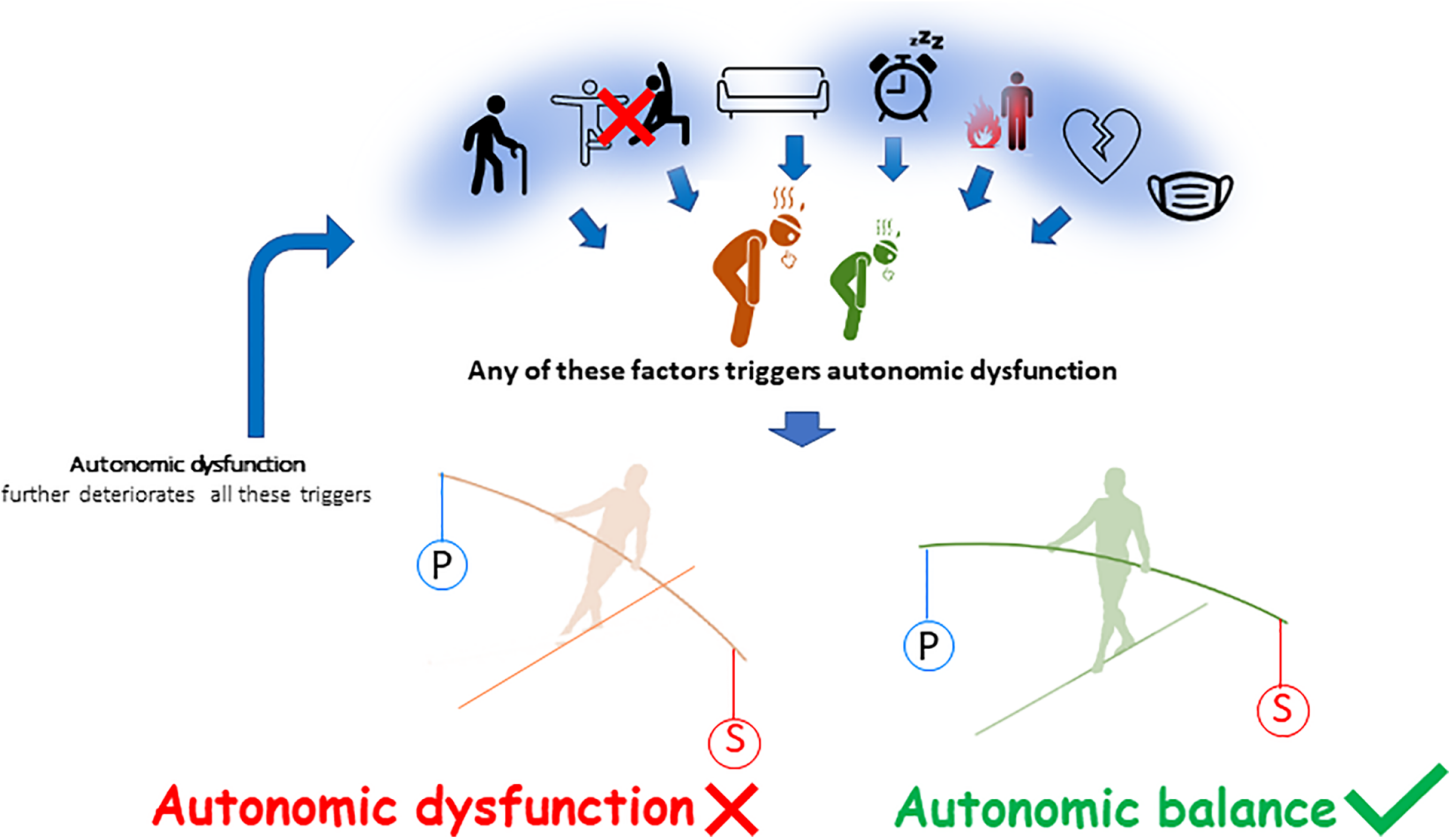
Determinants of autonomic dysfunction. From left to right, ageing, physical inactivity, sedentary behavior, sleep disorders, inflammation, cardiovascular diseases and more generally chronic disease may contribute to chronic fatigue and to a global decrease in autonomic function. There is a predominance in the sympathetic activity (S) to the detriment of the parasympathetic activity (P). The autonomic imbalance or autonomic dysfunction further increases fatigue (86).

Also, autonomic dysfunction, i.e., increased activity of sympathetic tone and/or less parasympathetic tone at rest, is associated with physical deconditioning and/or with chronic fatigue (82), since these two entities are very often linked. Whatever the mechanisms of fatigue, patients with fatigue exhibit signs of autonomic dysfunction, which may be increased by physical deconditioning (83–85) (Figure 3) (86). Cardiopulmonary exercise testing (CPET), that usually involve gas exchanges and kinetics of lactate production and removal measurements, allows to assess aerobic capacities in patients with chronic fatigue (87). The main cardiopulmonary parameters recorded are exercise duration (in min), maximal aerobic power (in W), maximal heart rate (in bpm), oxygen uptake (VO_2_, in L/min) at the first ventilatory threshold (VO_2_ at VT1) and at maximal exercise (VO_2_peak). Autonomic function is assessed by a resting ECG recording beforehand [with baroreflex sensitivity and heart rate variability (HRV) measurements] (81) and by heart rate recovery immediately following the CPET (88).

The analysis of the ANS is based on three indices: (i) spontaneous relative variations in blood pressure and R-R interval duration, recorded by measuring simultaneously a plethysmography blood pressure and ECG over a 15-min resting period in supine position. The increase or decrease in blood pressure and the corresponding lengthening or shortening response of the following R-R interval. The average slope of these regressions is considered as the index of baroreflex sensitivity (in ms/mmHg) (18, 81). For instance, Vigo et al. (89) suggested that reduced aerobic fitness, autonomic dysfunction, and feelings of fatigue represented the hallmark of the clinical phenotype of breast cancer survivors. These authors reported a smaller RR variance and baroreflex gain, suggesting an impaired parasympathetic performance, as compared to controls without fatigue (89). Also, Bertisch et al. (90) reported that baroreflex sensitivity was ∼20% lower in patients with sleep disorders compared to healthy controls. (ii) HRV indices measured from short (15 min) (91) or longer (*24h-ECG holter*) ECG recordings (81), using HRV analysis software [e.g., (92)]. The main time domain indices are the standard deviation of NN intervals, the root mean square of successive RR interval differences (RMSSD) and percentage of successive RR intervals that differ by more than 50 ms (pNN50) (Table 1). Fourier transform characterizes a signal by its frequency spectrum. This analysis allows to highlight low (LF) and high (HF) frequency ranges (Table 1). The LF/HF ratio allows to distinguish the balance between the sympathetic and the parasympathetic tones (81). Briefly, RMSSD, HF and PNN50 are regarded as specific indicators of the parasympathetic influence on the HR, whereas the standard deviation of NN intervals and LF components have a complex physiology that integrates both the sympathetic and parasympathetic components (81). For instance, Fagundes et al. (93) found that, in patients with breast cancer, fatigue was associated with elevated levels of norepinephrine (sympathetic response) and lower heart rate variability (parasympathetic response). In Rzepinski et al. (94), the R-R interval in normalized units was 25% lower in fatigued (assessed by Chalder Fatigue Scale questionnaire) people with multiple sclerosis vs. 30 controls patients who had no complaints of fatigue (32.7% vs. 44%). LF/HF-RRI was also 47% higher in these patients with fatigue in the same study. Moreover, HF was predicting the level of fatigue in multiple sclerosis patients in another study (95). Also, HRV kinetics during submaximal or maximal exercise may be predictive of aerobic fitness and exercise performance (96); (iii) Heart rate recovery represents reactivation of the parasympathetic tone, since vagal reactivation plays an integral part in reducing HR after exercise, especially during the first two minutes (88). For instance, heart rate recovery at two minutes was 23% lower in patients with sleep disorders comparted to healthy controls (35.5 vs. 46.5 bpm) (97). Similarly, HRV recovery following exercise occurs more rapidly in individuals with greater aerobic fitness (96).

**Table 1:** Main measures of heart rate variability time- and frequency-domains.

Parameter	Unit	Description
**Time-domain**		
SDNN	ms	Standard deviation of NN intervals
RMSSD	ms	Root mean square of successive RR interval differences
NN50	count	Number of successive differences in NN interval sequences greater than 50 ms
pNN50	%	Percentage of successive RR intervals that differ by more than 50 ms
HRmax−HRmin	bpm	Average difference between the highest and lowest heart rates during each respiratory cycle
**Frequency-domain**		
VLF peak	Hz	Peak frequency of the very-low-frequency band (0.0033-0.04 Hz)
VLF power	ms^2^	Absolute power of the very-low-frequency band (0.0033-0.04 Hz)
LF peak	Hz	Peak frequency of the low-frequency band (0.04–0.15 Hz)
LF power	ms^2^	Absolute power of the low-frequency band (0.04–0.15 Hz)
HF peak	Hz	Peak frequency of the high-frequency band (0.15–0.4 Hz)
HF power	ms^2^	Absolute power of the high-frequency band (0.15–0.4 Hz)
LF/HF	%	Ratio of LF-to-HF power

Interbeat interval, time interval between successive heartbeats; NN intervals, interbeat intervals from which artifacts have been removed; RR intervals, interbeat intervals between all successive heartbeats; LF: low frequency; HF: high frequency.

## Measuring objective fatigability: Why?

Discussing the protocols aiming at evaluating objective fatigability in response to physical activity is important because it may play a role in chronic fatigue. Indeed, we and others have recently evidenced a potential relationship between chronic fatigue and objective fatigability, provided objective fatigability is appropriately measured. As explained above, an extremely important observation is that in most studies in the field of chronic diseases, objective fatigability is measured during single-joint, isometric exercises. While those studies are valuable from a fundamental science point of view, their ecological validity does not allow to test the patients in real-life situations when the purpose is to search for a link with chronic fatigue.

Taul-Madsen et al. (40) compared isometric and concentric contractions fatiguing protocols and found that objective fatigability measured during a 2-minute sustained maximal isometric knee extension was not significantly associated with chronic fatigue whereas the concentric protocol was. Yet, in this paper, the concentric protocol consisted of 40 maximal isokinetic knee extensions at 30°/s, i.e., it was still far for activities of daily living. This is why it is worth reporting evidence coming from our group investigating the role of fatigability on fatigue, the former being measured with the innovative ergometer described above, i.e. closer to ecological conditions. We found that in people with multiple sclerosis, fatigability (together with depressive symptoms) influenced perception of fatigue (31). Similarly, in cancer survivors, tested 6 months to 5 years after the end of the treatment, we found that (in addition to V˙O_2_peak, TNF-*α*, age) fatigability explained 35% of the variance in cancer-related fatigue severity (30). Fatigability at a given submaximal workload was also lower in the fatigued group (Facit-F ≤ 34) than in the non-fatigued group (29). It was also found that the rapid impairments in fatigability in people with high level of cancer-related fatigue were primarily due to disturbances at the muscle level rather than at the central nervous system level.

**Figure 4:**
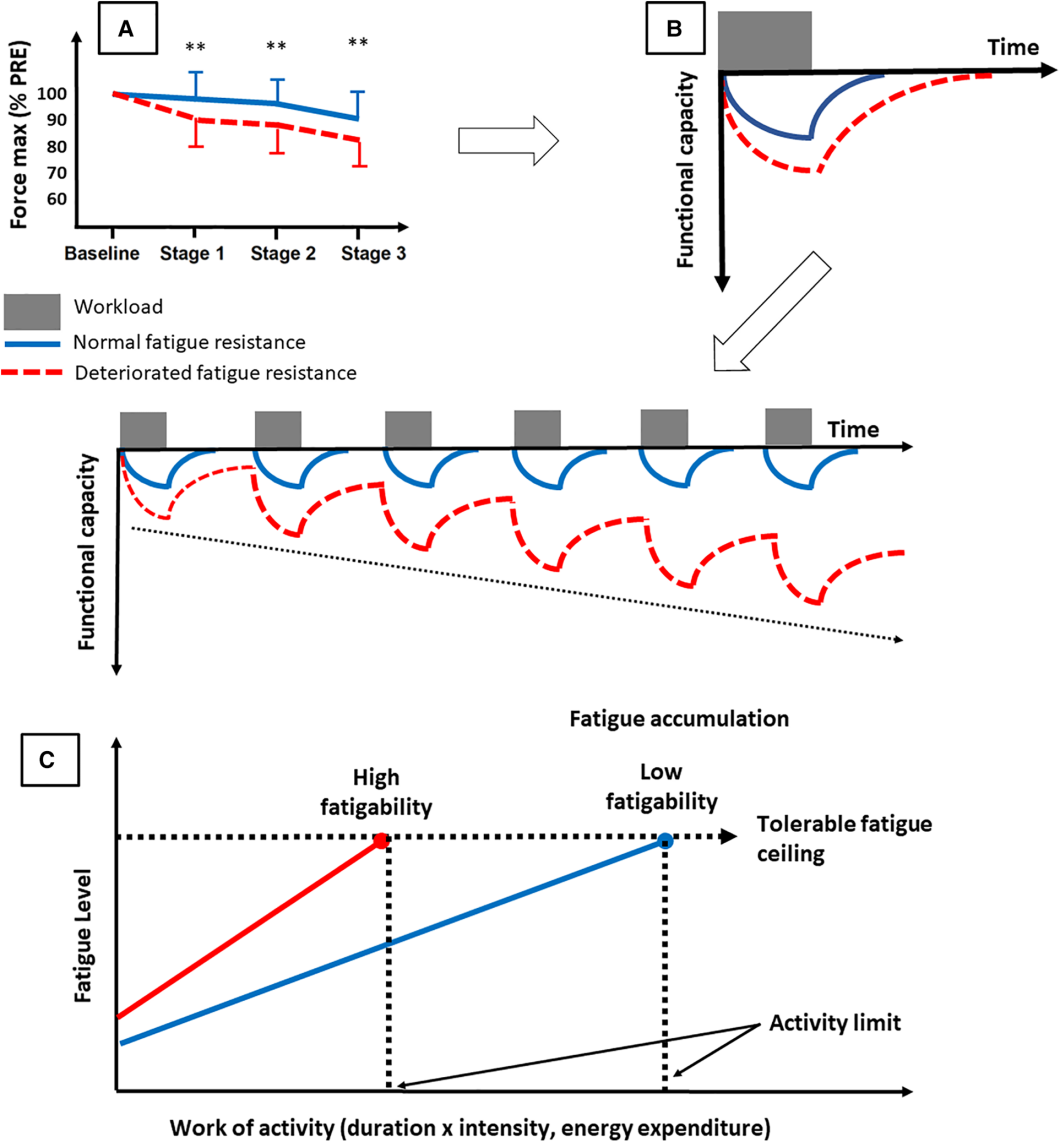
Tentative explanation of the consequences of deteriorated objective fatigability on fatigue. A deteriorated resistance to objective fatigability [**A**, adapted from (29)] may lead to a greater functional decline for a given task and more time to recover from daily life activities. The tasks performed in daily life could induce an accumulation of fatigue over time (**B**) or, more likely, could lead the persons to reach their ceiling of subjective fatigue earlier in the day [**C**, adapted from (97)]. This may force them to enter in the vicious circle of fatigue.

Overall, these studies show a lower resistance to objective fatigability, starting from low intensity exercise, in chronically fatigued patients. A tentative explanation is provided below. The lower resistance to fatigability (Figure 4A) (29, 98) leads to a greater functional decline for a given submaximal effort, especially if this is associated with slower VO_2_ kinetics (99). In this latter paper, our group shows that subjects with slow VO_2_ kinetics experience more peripheral fatigue, in particular more excitation-contraction coupling failure. Yet since this study was conducted using high intensity cycling, whether this applies to submaximal effort, such as walking, needs to be further investigated. The required tasks in daily life, hour after hour, day after day, could induce an accumulation of fatigue over time (Figure 4B) (100). The reality might be more like what is depicted in Figure 4C, where the people with lower resistance to objective fatigability would reach their ceiling of subjective fatigue earlier and thus be less active in their daily life (98). This could lead them to enter what is commonly referred to as the vicious cycle of fatigue: less physical activity leads to physical deconditioning and autonomic dysfunction, which in turn leads to more fatigue, which induces lower levels of physical activity. Recently, in long COVID-19 patients, physical deconditioning assessed by CPET was associated with fatigue and exercise intolerance as in myalgic encephalitis and/or chronic fatigue syndrome, fibromyalgia, and heart failure (87). In these different etiologies of fatigue, physical deconditioning is a major cause of reduced exercise capacity. Correlative data with cardiac autonomic measures may provide relevant insights into fatigue since autonomic dysfunction is also associated with reduced physical capacity (87). It must however be acknowledged that the experimental evidence showing impaired resistance to objective fatigability in patients with chronic diseases are relatively scarce. It is nevertheless worth mentioning that we recently showed that for a given amount of work completed, fatigability is higher when exercising above gas exchange threshold (101). This is possibly the case for many deconditioned patients.

**Figure 5:**
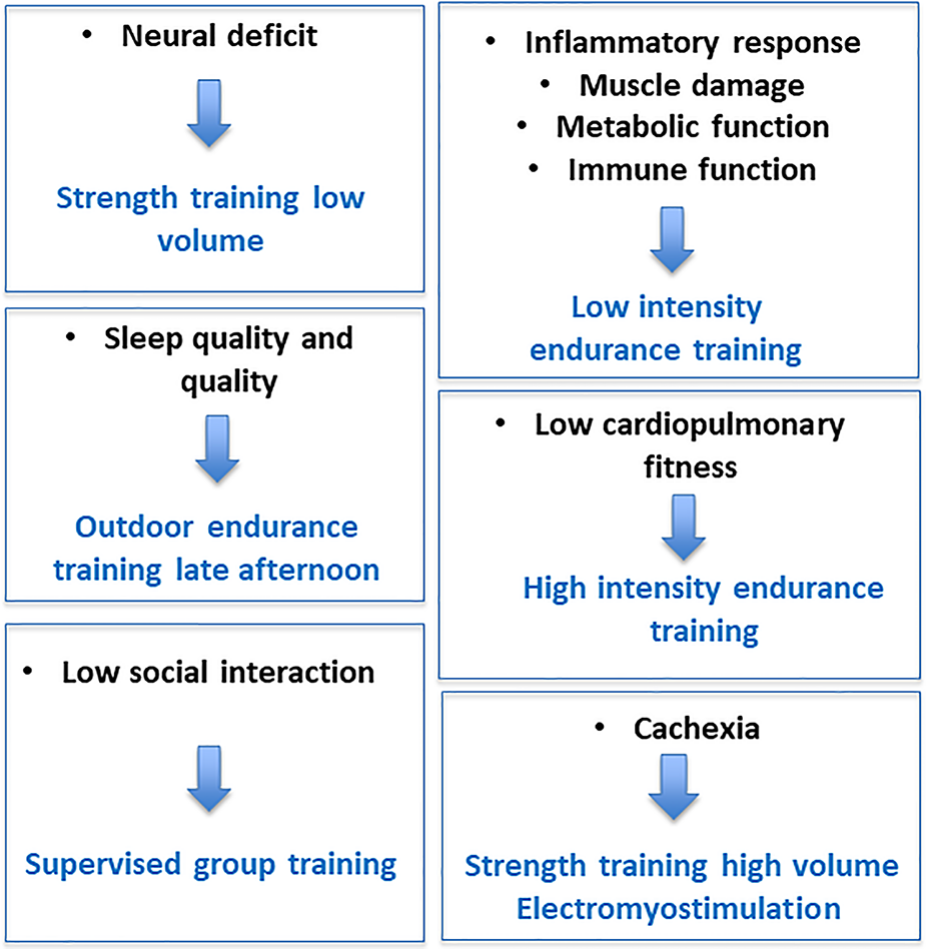
Examples of tailored exercise interventions.

To prevent the accumulation of fatigue and the autonomic dysfunction, interventions on recovery from fatigue *via* normalization of sympathetic/parasympathetic balance may be useful (96). Even if little is known about the mechanisms that associate autonomic dysfunction, physical deconditioning, and chronic fatigue in patients, regular exercise counterbalances these three entities (81). However, an important and still unanswered question is what type of exercise is best to fight fatigue? In cancer, results of meta-analyses indicate that aerobic exercise interventions are associated with statistically significant reductions in fatigue levels, whereas the effects are less clear for resistance exercises (102). According to the French National Cancer Institute, the optimal conditions under which physical activity is likely to reduce cancer-induced fatigue (type of exercise, intensity, duration and frequency, practice environment, etc.) remain debated. The treatment of fatigue is complex and needs to be individualized (103). This is quite normal given all the above, the complexity and the plurality of causes. Our team has proposed that a targeted intervention on the etiology of fatigue can optimize the effects of the program and decrease fatigue (104). Some examples are shown in Figure 5. In order to target the most beneficial exercises for individuals, it is necessary to consider at least key factors such as age, disease typology (e.g., type of cancer or type of multiple sclerosis), medical co-morbidities (e.g., overweight, diabetes), sports history and, most importantly, personal preferences (105). Yet, a better evaluation of fatigue etiology may allow to personalize the training intervention. In other words, despite some common characteristics of training are recognized, only an appropriate evaluation of fatigue will allow an individualized exercise intervention. In particular, the resting autonomic function assessment (pre-CPET) will aim to identify fatigue objectively, while the exercise physical capacity and ANS assessment (CPET) aim to analyze and understand the possible mechanisms responsible for fatigue and exercise intolerance (87). CPET and ANS assessments can also be used to define an individualized exercise intervention, e.g. by determining the intensity domains. We believe this is key in order to account for the complex, multifactorial nature of chronic fatigue.

It has been reported that post-exertional malaise is a significant challenge in patients with myalgic encephalomyelitis/chronic fatigue syndrome (106) and in cancer patients (107). The same observations have been made for long COVID patients with severe chronic fatigue (Facit-F score = 18) (108). These are the rare situations where evaluating fatigability may not be doable (in addition to the patients being not able to exercise due to their disability), particularly if the evaluation requests maximal exercise. In that case, assessment of objective fatigability after the first stage of the QIF test on the innovative ergometer may represent an option. It is also possible to adapt the evaluation with submaximal CPET (109). Also, the relevance of the evaluation of physical capacity and autonomic function goes well beyond the analysis of the mechanisms leading to fatigue. Associated physical capacity and ANS assessment is also used to (i) follow the evolution of fatigue (using repeated HRV and heart rate recovery measures) to further individualize exercise intervention (81) and (ii) compare a patient with an healthy group of the same age (110).

More importantly, whether exercise interventions are beneficial or counterproductive in these patients is questionable. It has been suggested that multidisciplinary rehabilitation programs, based on individualized and tailored exercise prescriptions, must be added to the continuum of care in long Covid patients (111). For all patients, but especially for patients suffering from post-exertional malaise, monitoring fatigue regularly during the intervention, for instance by considering autonomic dysfunction or subjective fatigue (112, 113), is mandatory to adjust the workload. As explained above, this could represent another fundamental way to individualize training intervention but still has to be validated scientifically.

## Conclusion

The present paper focused on some objective measurements that can be performed in fatigued patients, whether fatigue is due a specific chronic disease or is idiopathic. There is a need to appropriately measure objective fatigability and autonomic dysfunction so that the outcomes are both reproducible (in order to track the change with time) and relevant, i.e., as ecological as possible to be associated with chronic fatigue. The challenge for clinicians is to perform these valid tests in a time compatible with their professional schedule. Also, given the reduced exercise capacity among most fatigued patients, knowing the deconditioning can worsen when entering in the vicious circle of fatigue, CPET with additional ANS measures is recommended at the beginning of the training intervention to personalize the program. We also suggest that ANS outcomes, subjective fatigue assessments and regular objective fatigability measurements are useful measures to be included in interventional protocols for chronic fatigue in order to adjust the training load, particularly in the most fragile populations.
